# Nutriepigenetics and nutriepigenomics: Exploring the interactions between nutrition, epigenetics, and epigenomics in the pathogenesis of noncommunicable diseases

**DOI:** 10.1590/1678-4685-GMB-2025-0113

**Published:** 2026-07-17

**Authors:** Miguel Ángel Cáceres-Durán, Ândrea Ribeiro-dos-Santos

**Affiliations:** 1 Universidade Federal do Pará, Instituto de Ciências Biológicas, Laboratório de Genética Humana e Médica, Belém, PA, Brazil.; 2 Instituto Agronômico de Campinas, Centro de Citricultura “Sylvio Moreira”, Cordeirópolis, SP, Brazil.

**Keywords:** Nutriepigenetics, nutriepigenomics, noncommunicable diseases epigenetics, DNA methylation, ncRNAs

## Abstract

The complex interaction between nutrition, epigenetics, and epigenomics in non-communicable diseases (NCDs) such as cardiovascular diseases, obesity, type 2 diabetes mellitus (T2DM), and cancer highlights the crucial role of nutrition as an environmental factor influencing gene expression through epigenetic mechanisms such as DNA methylation, histone modifications, and regulation through non-coding RNAs (ncRNAs). The aim of this review is to explore the importance of interactions between nutrition-epigenetics interactions in the pathogenesis of NCDs and in the development of personalized prevention and treatment strategies. These nutriepigenetic and nutriepigenomic processes are fundamental for understanding the underlying molecular mechanisms of NCDs development. Diet plays a central role in modulating gene expression, and studies indicate how nutrients and bioactive compounds in food can directly affect epigenetic patterns, influencing the risk and progression of NCDs. For example, dietary effects on DNA methylation and miRNA expression have been associated with changes in susceptibility to cardiovascular diseases, obesity, and T2DM. Additionally, phytochemicals such as curcumin, genistein, quercetin, equol, among others found in certain foods can epigenetically modulate gene expression, playing a role in cancer prevention. The complexity of nutriepigenetic and nutriepigenomic systems highlights the need for a personalized approach to disease prevention and treatment. Understanding how diet influences epigenetic patterns can provide crucial insights for the development of more targeted and effective therapeutic strategies, ultimately underscoring the growing translational value of nutriepigenetics and nutriepigenomics in advancing precision medicine and informing population-level interventions, reinforcing their clinical, preventive, and societal impact in mitigating the burden of NCDs.

## Introduction

The interaction between genetics and the environment is essential for health and disease, where genes determine susceptibility, and diet influences their manifestation (Simopoulos, 2010). Within this context, nutrigenetics examines how genetic variation influences the response to specific nutrients, while nutrigenomics studies how nutrients and bioactive dietary compounds affect gene expression and metabolic pathways at the genomic level (Fenech et al., 2011; Carlberg et al., 2016).

Epigenetics, a term coined by Waddington in 1942, investigates the interactions between genes and their products in the manifestation of the phenotype. Today, it refers to phenotypic modifications not derived from changes in the DNA sequence, which are transmitted mitotically and/or meiotically (Armstrong, 2014). The epigenome includes chemical modifications to DNA that regulate gene expression, including DNA methylation, histone modifications, and non-coding RNAs (ncRNAs) (Wei et al., 2017; Maulani and Auerkari, 2020).

Building upon this framework, nutriepigenetics and nutriepigenomics have emerged as integrative fields linking nutrition and epigenetic regulation. Nutriepigenetics explores how food influences gene expression and how nutrition-induced epigenetic marks can be transmitted across generations without altering the DNA structure, shaping health and disease outcomes through epigenetic mechanisms (Cacabelos et al., 2025). Nutriepigenomics, in turn, focuses on the interaction between nutrition and the epigenome, recognizing the marked influence of nutrients on diverse epigenetic modifications and the likelihood that dietary components modulate key biochemical pathways via epigenetic mechanisms (de Luca et al., 2017).

The growing interest in epigenetic reprogramming underscores the potential of diet as a modifiable factor capable of shaping long-term health trajectories, thereby positioning nutriepigenetics and nutriepigenomics as promising approaches for disease prevention (Kenanoglu et al., 2022). Research in this area helps in understanding the molecular and epigenetic mechanisms involved in non-communicable chronic diseases (NCDs), contributing to personalized prevention and treatment strategies aligned with personalized medicine, promoting more effective approaches to public health challenges.

## Epigenetic mechanisms

As previously mentioned, among the various types of epigenetic modifications are DNA methylation, histone alterations, and ncRNAs (Wei et al., 2017; Maulani and Auerkari, 2020).

**Figure 1-:**
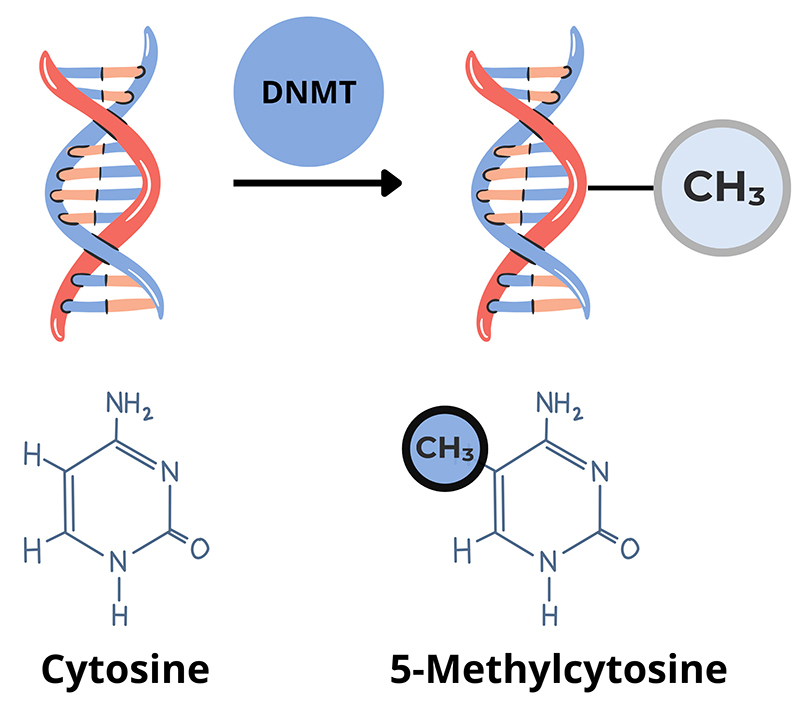
DNA methylation. DNA methylation refers to the process of adding methyl groups (CH_3_) to DNA, typically with the purpose of suppressing genetic transcription. This process involves the methylation of cytosine, resulting in the formation of 5-methylcytosine, which occurs at the fifth position of the pyrimidine ring. This modification is carried out by enzymes known as DNA methyltransferases, also referred to as DNMTs.

DNA methylation, a key epigenetic modification, involves adding a methyl group (CH_3_) to cytosine, primarily in CpG islands (Cáceres-Durán et al., 2023) (Figure 1). S-adenosylmethionine (SAM), the methyl donor, relies on nutrients like choline, betaine, methionine, and methyl-THF, which are interconnected with vitamins B6 and B12. Disruptions in these metabolic pathways affect DNA methylation and gene expression. A methyl-rich diet in early pregnancy is crucial for fetal development, as improper methylation increases disease and cancer risk. Some epigenetic modifications are inheritable and vital for embryogenesis (Tsalidou and Papaioannidou, 2010; Alavian-Ghavanini and Rüegg, 2018). Nutrients also influence DNA methyltransferases (DNMTs) and histone deacetylases (HDACs) (Kumar, 2015) (Table 1).

**Table 1 -:** Epigenetic modulation and biological activities of nutrients and bioactive compounds.

Type	Compound	Epigenetic Effect	Biological Activity	Source	Reference
Vitamin	Folate	DNA and histone methylation	DNA synthesis and repair, amino acid metabolism, neural tube development	Leafy greens, legumes, seeds, liver	Tsalidou and Papaioannidou, 2010; Alavian-Ghavanini and Rüegg, 2018; Durán et al., 2021
	Vitamin B12	DNA methylation	DNA synthesis, neurological function, fatty acids and amino acids metabolism	Meat, eggs	Adaikalakoteswari et al., 2015; Mahajan et al., 2019
	Vitamin D	DNA methylation, histone acetylation and methylation, regulation of miRNAs expression	Regulation of calcium and phosphorus metabolism, immune system modulation, cardiovascular health	Sun exposure, fatty fish, fortified foods	Escobedo-Monge et al., 2024; Gonzalez-Duarte et al., 2015; Pai *et al.*, 2013
Flavonoid	Quercetin	DNA methylation, histone acetylation and methylation, regulation of miRNAs and lncRNAs expression	Anti-oxidant, anti-angiogenic, anti-inflammatory, and anti-cancer effects	Onions, apples, berries	Reyes-Farias et al., 2019; Zhu et al., 2023
	Equol	DNA methylation, histone acetylation and methylation, regulation of miRNAs and lncRNAs expression	Antioxidant properties, estrogenic and anti-estrogenic effects, anti-inflammatory effects	Soy products	Hod et al., 2021; Mayo et al., 2019
	Apigenin	Regulation of miRNAs expression	Anti-inflammatory and anti-fibrosis activity	Parsley, celery, chamomile tea	Hou et al., 2021
	Isorhamnetin	DNA methylation	Metabolic regulation, anti-inflammatory and anti-cancer effects	Pears, almonds, olives	Matboli et al., 2021
Polyphenol	Resveratrol	Regulation of miRNAs expression	Antioxidant, anti-inflammatory, inhibition of proliferation and metastasis, induction of apoptosis	Grapes, red wine	Meng et al., 2020; Tomé-Carneiro et al., 2013
	Epigallocatechin-3-gallate	HAT inhibitor, DNMT inhibitor, histone methylation inhibitor	Anti-oxidant and anti-angiogenic activity	Green tea	Balasubramanian et al., 2010; Choi et al., 2009; Li et al., 2017
	Caffeic Acid	Regulation of miRNAs expression	Anti-oxidant and anti-inflammatory activity	Coffee, fruits, vegetables	Ding et al., 2023; Salem et al., 2019
	Curcumin	Regulation of miRNAs expression	Anti-inflammatory, antioxidant, anticancer, antibiotic, potential anti-aging effects	Turmeric (*Curcuma longa*)	Momtazi et al., 2016; Li et al., 2019; Liu et al., 2019; Hassan et al., 2019; Kronski et al., 2014; Yang et al., 2010
Fatty Acid	Butyrate	DNA methylation, histone acetylation and methylation, regulation of miRNAs expression	Metabolic regulation, anti-inflammatory and anti-cancer effects, gut barrier function	Fermented foods, fiber-rich foods	Bishop et al., 2017; Noureldein et al., 2020
	Eicosapentaenoic Acid (EPA)	DNA methylation	Lipid metabolism	Fatty fish	Ma and Ordovas, 2017
Saponin	Dioscin	Regulation of miRNAs expression	Antitumor, anti-inflammatory, hepatoprotective, and antiviral activities	Wild yam, fenugreek	Sun et al., 2021
Mineral	Sodium	DNA methylation, histone acetylation and methylation, regulation of miRNAs expression	Fluid balance, blood pressure regulation, nerve and muscle function, osmotic pressure	Salt, processed foods	Kotchen et al., 2013; Long et al., 2017; Ferrero et al., 2021; Liao et al., 2023
Ubiquinone	CoQ10	DNA methylation, histone acetylation and methylation, regulation of miRNAs expression	Energy production, antioxidant protection, cellular repair and regeneration, immune functions	Meat, fish, whole grains	Hargreaves et al., 2020; Alehagen et al., 2017
Sugar	Xylobiose	Regulation of miRNAs expression	Prebiotic activity, modulation of gut microbiota, anti-inflammatory activity	Plant cell walls, xylose-rich plants	Corrêa and Rogero, 2020; Singh et al., 2015

**Figure 2-:**
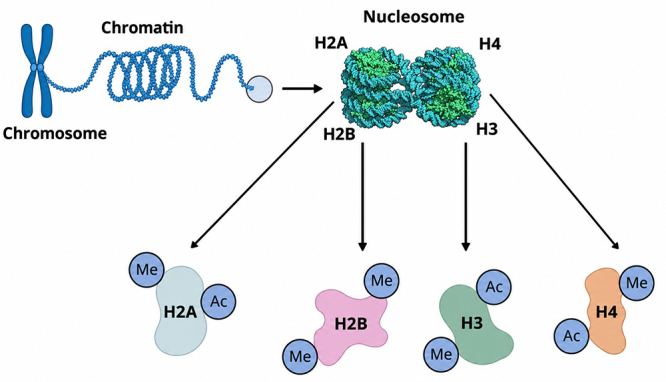
Chromatin structure and histone modifications on the N-terminal tails of histones. The eukaryotic genome is organized by the wrapping of DNA around histone octamers to form the basic units of chromatin, nucleosomes. The histone octamer consists of two copies each of histones H2A, H2B, H3, and H4. In addition to their globular domains, each of them possesses N-terminal tails that can undergo post-translational modifications, and all known acetylation and methylation modifications on lysine residues in each tail are highlighted.

In eukaryotic cells, DNA forms chromatin, with nucleosomes as functional units (Figure 2). Each nucleosome consists of a histone octamer (H2A, H2B, H3, H4) and 147 base pairs of DNA (Bannister and Kouzarides, 2011). Histone N-terminal tails undergo post-translational modifications (PTMs) like acetylation and methylation, affecting chromatin structure and gene expression (Lawrence et al., 2016; Zhao and Shilatifard, 2019).

**Figure 3-:**
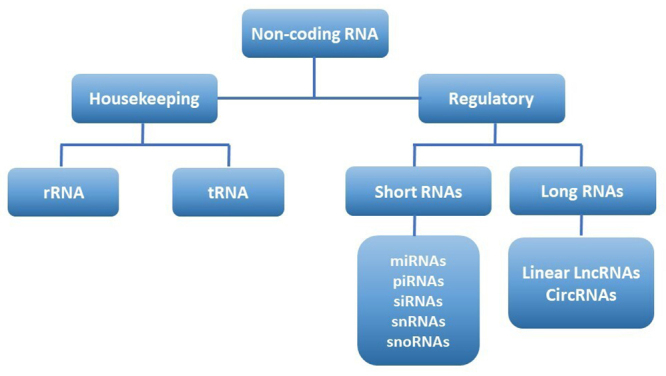
Functional classification of ncRNAs. ncRNAs are functionally classified into housekeeping ncRNAs and regulatory ncRNAs. Within the regulatory category, there are short ncRNAs (<200 nt) and long ncRNAs (>200 nt).

NcRNAs also play a significant role in epigenetics. Most of the genome is transcribed into ncRNAs, which regulate gene expression more than protein-coding genes (Katayama et al., 2005; Chadwick and Scott, 2013). Consequently, it is now believed that the degree of complexity of a species is more correlated with the number of ncRNAs than with the number of protein-coding genes (Taft et al., 2007). Mutations in ncRNA regions can contribute to disease development, including cancer, depending on their functional impact and biological context (Melton et al., 2015). NcRNAs can be classified as housekeeping or regulatory, based on their biological function (Figure 3), with regulatory ncRNAs further divided into short (<200 nt) and long (>200 nt) transcripts (Bhat et al., 2020). This includes long ncRNAs (lncRNAs), circular RNAs (circRNAs), microRNAs (miRNAs), small interfering RNAs (siRNAs), small nucleolar RNAs (snoRNAs), and PIWI-interacting RNAs (piRNAs) (Klinge, 2018; Ning and Li, 2018). MiRNAs, typically 19-25 nucleotides, regulate gene expression post-transcriptionally, influencing entire pathways (Ning and Li, 2018; Lu and Rothenberg, 2018). LncRNAs, longer than 200 nt, participate in transcriptional, post-transcriptional, and epigenetic regulation (Fang and Fullwood, 2016; Valenti et al., 2017; Xu et al., 2020a).

**Figure 4-:**
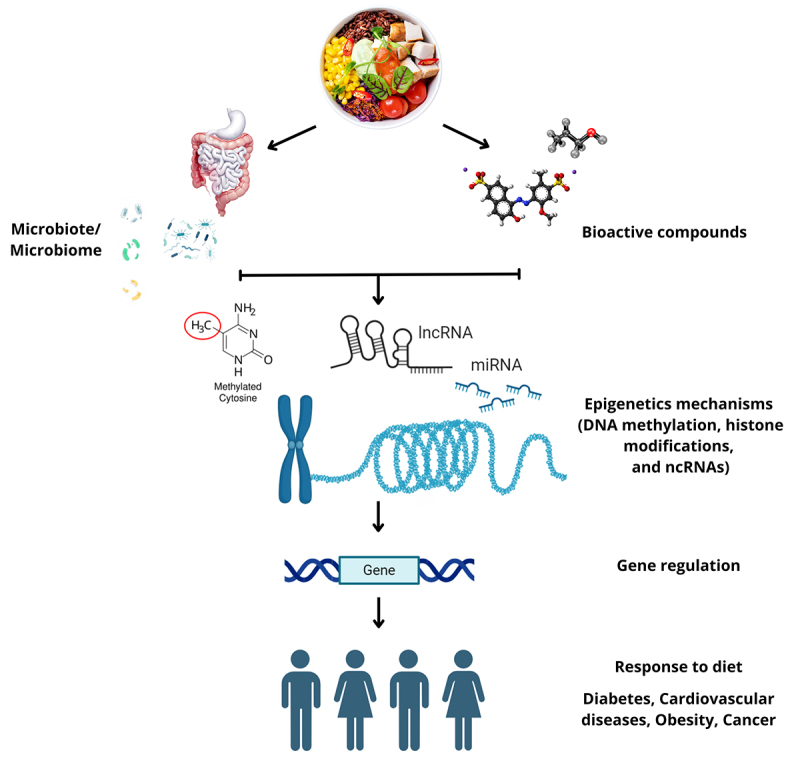
Interaction between nutriepigenetics, nutriepigenomics, and health. The diagram illustrates how diet affects our microbiota/microbiome and how bioactive compounds in foods (and the microbiome) participate in epigenetic mechanisms. Nutriepigenetics refers to how nutrients and bioactive compounds in foods can influence DNA methylation, histone modifications, and the regulation of ncRNAs. Nutriepigenomics, on the other hand, studies how these epigenetic changes at the genomic level can affect gene expression in response to diet. These changes in gene expression can be reflected in the phenotype, influencing the onset and progression of diseases such as diabetes, obesity, and cancer. The figure highlights the complexity and interconnection between nutrition, epigenetics, and health, emphasizing the importance of a balanced diet for the prevention and management of non-communicable chronic diseases.

Environmental factors, such as diet, influence epigenetic inheritance through germline cells (Bordoni and Gabbianelli, 2019). Diet provides substrates and cofactors for enzymes modifying DNA, RNA, and histones, impacting gene regulation (Vaziri and Dus, 2021) (Figure 4, Table 1). By modifying the activity of metabolic enzymes or the flow of metabolic pathways, the composition of the diet not only alters the availability of cofactors for these modifications but also regulates the binding of gene regulatory complexes to their substrates (Haws et al., 2020). It has been demonstrated that diet can modulate the profile of circulating miRNAs. Nutritional modulation of miRNA profiles highlights its role in gene regulation and chronic disease risk. Nutrimiromics explores how diet affects gene expression through miRNA-related processes (Quintanilha et al., 2017). Bioactive compounds like quercetin and curcumin modulate epigenetic activity, offering therapeutic potential, especially for cancer and metabolic diseases (Bordoni and Gabbianelli, 2019).

## Nutriepigenetics and cardiovascular diseases

Cardiovascular diseases (CVDs) remain the leading cause of mortality worldwide (Townsend et al., 2022), with their prevalence expected to increase in both developing and developed countries (Vasan and Benjamin, 2016). The pathophysiology of CVDs involves endothelial dysfunction, vascular inflammation, atherosclerosis, fibrosis, and thrombosis, with multiple disrupted pathways (Tabas, 2017). Vascular aging contributes to disease onset through remodeling and loss of flexibility, accumulating molecular damage markers such as telomere shortening, epigenetic alterations, and mitochondrial dysfunction (López-Otín et al., 2023).

Several environmental factors linked to CVDs are influenced by epigenetic mechanisms, including PTMs on histones, DNA methylation, and ncRNA-mediated chromatin remodeling. Despite extensive research on the effects of diet and physical activity on CVD outcomes (Wallace et al., 2018; Siddiqui et al., 2024), limited evidence exists on how dietary components modulate the epigenome of aged vascular cells.

Excessive nutrient intake is linked to CVDs, metabolic syndrome, and insulin resistance. High carbohydrate and lipid consumption raise acetyl-CoA levels, altering chromatin structure and suppressing autophagy. Fatty acids like α-linolenic acid, eicosapentaenoic acid (EPA), and docosahexaenoic acid (DHA) affect DNA methylation of CVD-related genes such as apolipoproteína E (*APOE*), interleukin-6 (*IL6*, and *ABCA1*), while obesity reduces perilipin 1 (*PLIN1*) expression (Kalea et al., 2018).

Vitamin B12 deficiency induces hypomethylation of sterol regulatory element binding protein 1 (*SREBF2*) and low-density lipoprotein receptor (*LDLR*) promoter regions, leading to increased cholesterol biosynthesis in adipose tissue (Adaikalakoteswari et al., 2015). Several CpG methylation sites and genes significantly contribute to heart and blood vessel diseases. Genome-wide association studies confirm their downstream effects on cardiovascular phenotypes, influencing vascular markers such as blood pressure and cardiac function (Krolevets et al., 2023).

Natural compounds influence circulating miRNA expression regardless of dietary patterns. Strong associations were observed between dietary components and miRNA profiles in omnivores, vegetarians, and vegans, with sodium, cholesterol, vitamin D, and vitamin E showing the strongest correlations (Ferrero et al., 2021). In males supplemented with vitamin D, a weak correlation was found with miR-532-3p, a biomarker for pre-diabetes and colorectal cancer (Jorde et al., 2012; Zhao et al., 2021; Mathur and Rani, 2023). It also plays a role in atherosclerotic plaque formation (Zhao *et al.*, 2021).

Sodium, mainly consumed as salt (NaCl), alters 16 plasma miRNAs. MiR-23a-3p, abundant in plasma and extracellular vesicles, increases with sodium intake and is enriched in vascular endothelial cells (Kotchen et al., 2013; Rie et al., 2017). It regulates cardiomyocyte growth, inhibits an anti-hypertrophic protein, and affects apoptosis (Long et al., 2017). Elevated miR-23a-3p levels correlate with postoperative atrial fibrillation and may predict coronary lesions (Di et al., 2015). Sodium-driven miRNA regulation may explain the dietary salt-cardiac function link (Ferrero et al., 2021). In addition, innate immune antigen-presenting cells contribute to the rapid rise in blood pressure triggered by excessive sodium intake. Emerging evidence suggests that epigenetic reprogramming, accompanied by transcriptional and metabolic shifts, enables these innate immune cells to mount sustained responses upon repeated sodium-related stimuli (Mutchler et al., 2025). In salt-sensitive rats, transient high salt intake increased H3K4me1 at the *NF-κB p65* promoter, triggering inflammation, hypertension, and kidney damage (Liao et al., 2023).

Extra virgin olive oil modulates cardiovascular-related miRNAs postprandially (Daimiel et al., 2020). Let-7e-5p decreases after consumption, while the miR-17-92 cluster rises with low-to-moderate polyphenol levels, suggesting a role in fatty acid metabolism and cardiovascular benefits (Daimiel *et al.*, 2020).

In a study with 443 elderly individuals (Alehagen et al., 2017), four-year supplementation with selenium and coenzyme Q10 (CoQ10) significantly altered 70 miRNAs, indicating cardioprotective effects. Notable increases were seen in miR-29b-3p, linked to cardiac extracellular matrix regulation, and miR-30e-5p, potentially protective against heart failure. Elevated miR-19a-3p expression suggested additional protective effects. Another study (Tarallo et al., 2014) highlighted differential miR-92a expression in plasma and fecal samples among individuals with different dietary habits, showing higher levels in vegetarians and vegans compared to omnivores.

Regarding cholesterol metabolism, miR-144-3p, miR-374a-5p, miR-1277-5p, and miR-1273c exhibited inverse correlations. MiR-144-3p was the most significantly inversely related to cholesterol, targeting ATP-binding cassette transporter A1 (*ABCA1*), known to enhance atherosclerosis and inflammation (De Aguiar *et al.*, 2013; Hu et al., 2014).

A study in mice demonstrated that genistein, a flavonoid found in various plants, inhibits vascular endothelial cell inflammation and reduces chronic inflammatory responses by downregulating miR-21. This mechanism is closely associated with *NF-κB p65* activity, suggesting potential anti-inflammatory therapeutic applications (Xie et al., 2021)

Summarizing, nutrients and bioactive compounds can influence gene expression, DNA methylation, and miRNA profiles, affecting cardiovascular disease development. This emphasizes the potential of dietary interventions to reduce cardiovascular risk and the need for further research to understand these complex interactions. Personalized nutrition, tailored to individual genetic and epigenetic profiles, can optimize dietary recommendations for preventing or managing CDVs. It helps identify specific bioactive compounds that modulate epigenetic marks beneficially, enhancing intervention effectiveness while minimizing adverse effects. This approach paves the way for precision medicine in cardiovascular disease management.

## Nutriepigenetics and metabolic diseases - obesity and diabetes

Obesity is a complex, multifactorial disease characterized by excessive fat accumulation with negative health effects. Its prevalence continues to rise, fueling a global epidemic with no signs of decline. Elevated body mass index (BMI) increases the risk of non-communicable diseases, including diabetes, cardiovascular diseases, and musculoskeletal disorders, significantly reducing quality of life and life expectancy (Lin and Li, 2021). However, BMI alone has limitations as a diagnostic tool. Recent evidence supports a refined classification distinguishing preclinical obesity, which increases future health risks, from clinical obesity, a chronic systemic disease directly linked to excess fat accumulation (The Lancet Diabetes and Endocrinology, 2025).

Type 2 diabetes mellitus (T2DM) is a chronic, multifactorial disorder often requiring multiple medications to regulate blood glucose. This endocrine-metabolic condition poses severe health risks, with global prevalence projected to reach 642 million by 2040 (Zhou et al., 2018). T2DM arises from inadequate insulin secretion by pancreatic β-cells, while α-cells frequently exhibit excessive glucagon production (Davegårdh et al., 2018).

Metabolic diseases like obesity and T2DM have prompted studies on epigenetic inheritance of nutritional risk, mainly in rodents. Adverse perinatal environments affect offspring phenotypes. Rats under 50% calorie restriction for 50 generations developed obesity-related metabolic disturbances linked to Ins2 promoter epigenetic modifications, which persisted for two generations despite restored nutrition (Hardikar et al., 2015).

Epigenetic modifications are crucial in the transmission of phenotypes and the predisposition to complex human diseases, such as obesity and T2DM. Methylation patterns can be inherited or influenced by environmental factors (Silva-Ochoa et al., 2023). Research has demonstrated that genetic variations and polymorphisms can affect DNA methylation through methylation quantitative trait loci (mQTLs) (Grundberg et al., 2013; Bell et al., 2011). Methylation in promoter/enhancer regions suppresses transcription, whereas methylation in gene bodies promotes elongation and alternative splicing (Silva-Ochoa *et al.*, 2023).

Several studies have investigated the importance of DNA methylation and other epigenetic elements in pancreatic islet function in different models (Yang et al., 2011; Volkov et al., 2017; Davegårdh et al., 2018). Initial studies in human islets investigated key genes related to β-cells and cellular metabolism. They found that the promoters of the insuline (*INS)* (Yang *et al.*, 2011), pancreatic and duodenal homeobox 1 (*PDX1)* (Jonsson et al., 1994), peroxisome proliferator-activaed receptor gamma coactivator 1-alpha (*PPARGC1A*) (Wu et al., 2016), and glucagon-like peptide-1 receptor (*GLP1R*) (Hall et al., 2013) genes were hypermethylated in islets from donors with T2DM compared to non-diabetics. Additionally, an increase in DNA methylation was related to reduced mRNA expression of the corresponding gene in diabetic islets, along with higher levels of glycated hemoglobin (HbA1c). This suggests a potential involvement in β-cell dysfunction in T2DM. Also, high glucose levels were observed to directly increase DNA methylation of *PDX1* and *INS* in clonal β-cells (Yang *et al.*, 2011, 2012).

Global DNA methylation and hydroxymethylation correlate with waist circumference, glucose, cholesterol, and triglycerides, serving as potential metabolic biomarkers. Methylation patterns vary by obesity treatment (diet or surgery) (Nicoletti et al., 2016). Maternal overfeeding during pregnancy alters global DNA methylation, increasing obesity risk in offspring (Sharp et al., 2015).

Obese individuals showed increased CpG methylation and reduced *PLIN1* expression, affecting triglyceride lipolysis (Bialesova et al., 2017). Genome-wide methylation analysis revealed hypermethylation in peroxisome proliferator activated receptor gamma (*PPARG*)*,* heart and neural crest derivatives expressed 2 (*HAND2*)*,* homeobox C6 (*HOXC6*)*,* sorbin and SH3 domain-containing protein 2 (*SORBS2*)*,* CD36 molecule (*CD36*)*,* and claudin-1 *(CLDN1*) promoters, potentially impairing adipogenesis, triglyceride uptake, and insulin sensitivity (Keller et al., 2017).

High-fructose diets in animals induce peroxisome proliferator-activated receptor alpha (*PPARα*) and carnitine palmitoyltransferase 1A (*CPT1A*) promoter hypermethylation, reducing lipid β-oxidation and leading to metabolic syndrome (Munetsuna et al., 2019). Fructose consumption also alters hypothalamic and hippocampal methylation, impairing memory. DHA supplementation reverses these effects, improving cognition and normalizing histone methylation and acetylation levels (Meng et al., 2016).

Butyrate, a short-chain fatty acid, supports intestinal health by reducing inflammation and oxidative stress. In MKR mice with T2DM, butyrate treatment restored inflammatory markers, reduced reactive oxygen species (ROS), and alleviated colon inflammation. It counteracted dysbiosis, characterized by low butyrate levels and increased HDAC3 activity, suggesting its potential to mitigate diabetic complications (Noureldein et al., 2020).

Changes in histone-modifying enzymes are linked to obesity, influencing gene expression related to adiposity and metabolism. Key modifiers include HDACs and lysine demethylase 3A (*KDM3A*), a histone 3 lysine 9 (H3K9)-specific demethylase (Mahmoud, 2022). Histone modifications regulate adipogenesis-related genes like CCAAT/enhancer-binding protein beta (*C/EBPβ*)*, CCAAT/enhancer-binding protein alpha* (*C/EBPα*)*, preadipocyte factor 1* (*PREF-1*)*, adipocyte protein 2* (*AP2*)*,* and *PPARγ* (Zhang et al., 2012). Histone acetylation affects appetite-regulating genes, with high-fat diets reducing H3K9 acetylation at proopiomelanocortin (*POMC*) and increasing it at neuropeptide Y (*NPY*). Similarly, increased H3K9 and H3K18 acetylation at tumor necrosis factor (*TNF*) and C-C motif chemokine ligand 2 (*CCL2*) genes may contribute to inflammation (Mikula et al., 2014). Conversely, caloric restriction and weight loss can reverse these changes, increasing H4 acetylation and GLUT4 expression in adipose tissues (Wheatley et al., 2011). These findings highlight histone modifications as crucial epigenetic regulators of adipogenesis, potentially influencing obesity onset and progression.

Additionally, folic acid (vitamin B9) deficiency is associated with anemia, obesity, and epigenetic alterations, such as hypomethylation of LINE-1 nuclear elements, increasing cancer and mental illness risks. However, folic acid supplementation during pregnancy can reverse these epigenetic changes (Altobelli et al., 2013; Agodi et al., 2015; Li et al., 2018; Mahajan et al., 2019). Similarly, vitamin D plays an important epigenetic role by modulating DNA methylation and histone modifications and by supporting mitochondrial function-an effect particularly relevant to obesity, given the central role of mitochondrial dysfunction in altered energy balance and adiposity (Goss and Voruganti, 2025).

LncRNAs respond to nutritional changes in metabolic tissues like the liver, adipose tissue, and muscle. Regulatory mechanisms triggered by fasting, feeding, and high-fat diets involve complex interactions with DNA, RNA, and proteins. Understanding these responses is key to addressing metabolic diseases. Additionally, exploring gender bias in lncRNA regulation may reveal sex-based disparities in metabolic diseases (Lovell and Anguera, 2022).

During fasting, blood glucose levels drop, prompting the body to mobilize energy reserves, with certain lncRNAs increasing expression to regulate gluconeogenesis and maintain energy homeostasis. In contrast, high-fat diets elevate fatty acids and lipids, modulating lncRNA expression involved in lipogenesis and adipocyte differentiation. Some lncRNAs may decrease to regulate metabolic pathways linked to excess fat metabolism (Lovell and Anguera, 2022).

Circulating miRNAs are potential biomarkers for diagnosing and predicting T2DM and its complications. Key miRNAs include miR-126, miR-21, miR-320, miR-33a/b, let-7, miR-205-5p, and miR-20, which inhibit the insulin signaling pathway (Feng et al., 2016; Corona-Meraz et al., 2019; Gentile et al., 2019 *),* miR-33a and miR-33b target insulin receptor substrate 2 (*IRS2*)*,* sirtuin 6 (*SIRT6*)*,* and AMP-activated protein kinase (*AMPK*), regulating insulin and glucose homeostasis (Corona-Meraz *et al.*, 2019). In obesity, miR-223 reduces pro-inflammatory macrophage infiltration into adipose tissue. Mice lacking miR-223 on a high-fat diet show increased inflammation. Obese patients exhibit an inflammatory profile with reduced regulatory T cells and increased CD8+ T cells, cytokine production, and miRNAs from immune cells that suppress pro-inflammatory responses (Nishimura et al., 2009; Zhuang et al., 2012; Torri et al., 2017).

It has been shown that consumption of grape extract rich in resveratrol (RVT), a natural polyphenolic compound found in various plants, increases the expression of miR-21, miR-663, miR-30c2, and miR-181b, while decreasing miR-155 and miR-34 in hypertensive individuals with T2DM (Tomé-Carneiro et al., 2013). MiR-21 promotes cell survival and reduces inflammation by suppressing phosphatase and tensin homolog (*PTEN*), activating the PI3K/Akt pathway (Chawra et al., 2024). MiR-663 reduces inflammatory cytokines and oxidative stress, improving vascular function by targeting jun B proto-oncogene, AP-1 transcription factor subunit (*JunB*) and jun D proto-oncogene, AP-1 transcription factor subunit (*JunD*), and lowering miR-155 (Michaille et al., 2021). MiR-30c2 enhances insulin sensitivity and lipid profiles, while miR-181b improves endothelial function and reduces inflammation (Tomé-Carneiro *et al.*, 2013; Shantikumar et al., 2014).

Caffeic acid, a phenolic compound, reduces miR-636 expression associated with diabetic nephropathy in rats (Salem et al., 2019), while xylobiose, a disaccharide, regulates miR-122a and miR-33a, improving hepatic gene expression and reducing inflammation and oxidative stress (Lim et al., 2016; Corrêa and Rogero, 2020). In pre-diabetic individuals consuming pistachios (57 g/day) for four months, plasma miR-15, miR-21, miR-29b, and miR-126 increased, while miR-192 and miR-375 decreased, suggesting a role in glucose metabolism and T2DM progression (Hernández-Alonso et al., 2017). Additionally, apigenin and dioscin influence miRNA expression, offering potential therapeutic strategies for T2DM (Ohno et al., 2013). Apigenin’s protective effect in diabetic nephropathy is linked to the miR-423-5p-USF2 axis (Hou et al., 2021), while dioscin enhances the inhibitory effect of miR-125a-5p on STAT3 signaling, improving glycolipid metabolism in T2DM (Xu et al., 2020b).

Understanding the intricate interplay between diet, epigenetics, and metabolic diseases such as obesity T2DM underscores the potential of dietary interventions in managing and preventing these conditions. These findings highlight the critical need for further research to explore the complex relationships between nutrition, epigenetics, and chronic diseases, paving the way for the development of personalized prevention and treatment strategies. By tailoring dietary recommendations and interventions based on individual epigenetic profiles, it is possible to enhance the efficacy of treatments, reduce the risk of disease progression, and ultimately improve public health outcomes in the fight against obesity and T2DM.

## Nutriepigenetics and cancer

Cancer is a disease characterized by the rapid growth of abnormal cells that can spread to other parts of the body, being one of the leading causes of death in the Americas. It is projected that cancer mortality will increase to 2.1 million by 2030. Early detection and appropriate treatment can increase the chances of cure in many cases (PAHO, 2025).

In addition to its aggressive proliferation, cancer is marked by profound alterations in cellular metabolism. In normal cells, energy is generated through mitochondrial respiration requiring oxygen, but in cancer cells, lactate is produced from glucose metabolism regardless of oxygen availability, known as the Warburg effect. This metabolic shift impacts epigenetics by altering acetyl coenzyme A and other molecules essential for epigenetic enzymes (Joseph et al., 2010). Continuous glucose uptake by cancer cells is linked to proinflammatory gene expression, such as interleukin-8 (Rofstad et al., 2006). Epigenetic dysregulation in cancer includes global hypomethylation and focal hypermethylation in CpG islands, leading to gene silencing and abnormal gene expression. These methylation patterns are tissue- and cell-specific, with variations among cancer patients (Stirzaker et al., 2016).

Given these epigenetic disruptions, attention has turned to the potential modulators of epigenome, particularly nutrition. Although nutrition and dietary factors have been associated with cancer risk, the idea that epigenetics serves as the mechanistic link between the two is not yet fully elucidated. Studies in animals have shown strong associations between multiple dietary factors and significant changes in the epigenome, many of them effects of maternal nutrition on methylation status in offspring; however, studies in humans have produced inconsistent results. It is assumed that nutritional components likely influence an individual’s cancer risk, and the mechanism by which cancer risk is affected is likely through epigenetic modification of an individual’s genome (Sapienza and Issa, 2016).

Expanding on this hypothesis, it becomes increasingly clear that nutrition may also exert indirect effects on the establishment or maintenance of epigenetic modifications. Both calorie restriction and calorie excess have effects on DNA methylation, and both are considered to have opposite effects on the rate of biological aging. Calorie excess, using high BMI as an indicator of calorie excess, is a risk factor for various types of cancer, and multiple changes in DNA methylation are associated with BMI (Horvath et al., 2014; Demerath et al., 2015).

In this context, specific dietary components have been shown to directly influence methylation status and other epigenetic marks. For instance, it was found that leafy greens and folate reduce gene methylation in lung cancer, while multivitamins also decrease methylation (Stidley et al., 2010). Nutrients like EGCG from green tea and annurca apple polyphenols can reverse methylation and reactivate genes such as hMLH1 in cancer models (Raganelli, 2018).

Among micronutrients, vitamin D regulates miRNA gene transcription by binding to the vitamin D receptor (VDR) motif in miRNA gene promoters, influencing miRNA maturation and processing genes like Drosha and Dicer (Giangreco and Nonn, 2013). It induces miR-498, which suppresses human telomerase reverse transcriptase (*TERT*) and inhibits ovarian cancer cell growth (Kasiappan et al., 2012). In cervical cancer, calcitriol treatment modulates miRNA expression, including upregulating miR-498 and miR-22, and downregulating miR-3921 (Gonzalez-Duarte et al., 2015). In breast cancer, vitamin D reverses stress-induced miRNA expression, enhancing cancer cell susceptibility to natural killer cells (Peng et al., 2016). In prostate cancer, it upregulates miR-21, miR-22, miR-29a/b, miR-134, miR-106b, miR-100, and miR-125b, while downregulating miR-17/92 cluster members and promoting cell cycle arrest (Wang et al., 2011, 2013). In colorectal cancer, calcitriol upregulates miR-627 and miR-22, which regulate histone methylation and suppress target gene expression (Padi et al., 2013). It also affects bladder cancer cell lines, upregulating miR-17, let-7a, miR-1201, miR-22, miR-96, and miR-125 in different cell lines (Ma et al., 2015). In lung cancer, calcitriol upregulates let-7a-2, promoting anti-proliferative effects (Guan et al., 2013). Vitamin D induces miR-145 in gastric cancer, inhibiting cell proliferation (Chang et al., 2015). In leukemia, it downregulates miR-181a/b, miR-17-5p/20a/106a, miR-125b, and miR-155, and upregulates miR-26a and miR-32, affecting the genes encoding the c-Myc and BIM proteins (Wang *et al.*, 2009; Iosue et al., 2013; Salvatori et al., 2013). Calcidiol treatment increases susceptibility to natural killer cells by downregulating miR-302c and miR-520c, affecting genes in the natural killer group 2 member D (*NKG2D*) ligand pathway (Min et al., 2013). In melanoma, miR-125b expression is inversely related to VDR levels, suggesting its role in VDR regulation and vitamin D resistance (Essa et al., 2010).

Carotenoids and polyphenols, such as resveratrol (RVT), have anticancer properties, influencing DNA methylation and epigenetic mechanisms. Polyphenols can reverse adverse epigenetic changes, reactivating tumor suppressors and antioxidant genes while inhibiting pro-inflammatory and cancer-promoting genes, highlighting their potential in cancer prevention and treatment (Lorenzo et al., 2022). Among these, RVT demonstrates antitumor effects by influencing the HDAC pathway, notably in prostate cancer, where it reactivates *PTEN*, normally inhibited by the MTA1/HDAC complex. This leads to the activation of pro-apoptotic genes like BCL2 associated X, apoptosis regulator (*Bax*) and cyclin dependent kinase inhibitor 1A (*CDKN1A*), inducing programmed cell death (Pervaiz, 2003; Dhar et al., 2015; Singh et al., 2017). RVT also regulates cell cycle genes, promoting apoptosis and inhibiting antiapoptotic genes in colon and colorectal cancer, while suppressing NF-κB activity to reduce tumor progression (Singh *et al.*, 2010; Firestein et al., 2008). Supporting its broad antitumor potential, RVT also exerts antiproliferative effects in hepatoma cell lines in a dose-dependent manner, inhibiting HDAC families and hyperacetylating histones (Venturelli et al., 2013). Additionally, RVT induces apoptosis in osteoblastoma cells and attenuates androgen receptor-mediated proliferation in breast cancer by activating sirtuin 1 (*SIRT1*) (Li et al., 2009; Zhang et al., 2016).

Similarly, curcumin is a bioactive polyphenol with antibiotic, anticancer, and potential anti-aging effects. It regulates gene expression by affecting miRNAs, such as overexpressing miR-181b to inhibit breast cancer metastasis (Kronski et al., 2014), and miR-15a/miR-16 to induce apoptosis in MCF-7 cells (Yang et al., 2010). Curcumin also regulates miR-21 and miR-34a to inhibit tumor growth and metastasis (Momtazi et al., 2016) and affects pancreatic cancer cells by modulating miR-22 and miR-199a expression (Sun et al., 2008). It may influence osteogenesis through miR-126a-3p (Li et al., 2019) and has dose-dependent effects on miRNA regulation in diseases such as breast cancer, nasopharyngeal carcinoma, and lung cancer (Hassan et al., 2019; Liu *et al.*, 2019).

Genistein, another dietary polyphenol, also modulates miRNA expression, notably suppressing human uveal melanoma and murine chronic lymphocytic leukemia cells through miR-16 (Salerno et al., 2009). Moreover, polyunsaturated fatty acids affect various miRNAs like let-7d, miR-15b, miR-107, miR-191, and miR-324-5p, with fish oil providing protection against carcinogen-induced miRNA dysregulation in animals (Davidson et al., 2009). Beyond miRNA modulation, genistein also affects key signaling and metabolic pathways. It inhibits NF-κB activation via Akt, Notch, or P53, upregulates Cyclin-Dependent Kinase Inhibitors (CDKIs), regulates gluconeogenesis through the mTOR pathway, and suppresses Wnt signaling by reducing cytoplasmic β-catenin accumulation (Javed et al., 2021).

Quercetin, a flavonoid glycoside, similarly exerts anticancer effects by modulating oncogene expression, promoting apoptosis, and affecting tumor angiogenesis through pathways like Wnt/β-catenin, PI3K/Akt/mTOR, and MAPK/ERK1/2 (Reyes-Farias and Carrasco-Pozo, 2019). It also influences hepatocyte metabolism and DNMT activity, modulating Nrf2/HO-1 and p38/STAT1/NF-κB pathways to reduce inflammation and DNA methylation, underscoring its hepatoprotective potential (Liu et al., 2015; Kanwal et al., 2016).

Further, quercetin plays a key role in DNA demethylation processes. It reverses hypermethylation by demethylating cyclin-dependent kinase inhibitor 2A (*CDKN2A*/*p16INK4a*) gene in colon cancer (Tan et al., 2009) and induces apoptosis in esophageal cancer by enhancing p16INK4α expression (Zheng et al., 2014). In bladder cancer, it reduces DNA methylation of ras association domain family member 1 (*RASSF1A*), *CDKN2A*, and estrogen receptor genes (Ma et al., 2006; Alvarez et al., 2018), and in cervical cancer, it inhibits HDAC activity, leading to DNA hypomethylation (Kedhari et al., 2019).

Moreover, quercetin modulates miRNA expression across several cancer types. In lung cancer, it increases miR-16 and decreases claudin-2 expression (Sonoki et al., 2015), and it prevents hexavalent chromium-induced cancer by targeting the miR-21-PDCD4 pathway (Pratheeshkumar et al., 2010; Zhang et al., 2012). In breast cancer, it enhances miR-146a expression, promoting apoptosis via cleaved-caspase-3 and downregulating the epidermal growth factor receptor (*EGFR*) (Tao et al., 2015). In pancreatic ductal adenocarcinoma, quercetin inhibits tumor growth by upregulating miR-let-7c and downregulating miR-200b-3p, thus reducing cancer aggressiveness. When combined with other polyphenols, it enhances miRNA regulation-for example, by inducing miR-let-7a and suppressing kirsten rat sarcoma viral oncogene homolog (*KRAS*) (Appari et al., 2014; Nwaeburu et al., 2016). Additionally, in colorectal cancer, the combination of quercetin and RVT reduces oncogenic miR-27a, increasing zinc finger and BTB domain containing 10 (*ZBTB10*) expression and downregulating Sp transcription factors (Del Follo-Martinez et al., 2013). Quercetin enhances miR-146a in colon cancer (Prendecka-Wróbel et al., 2023; Li et al., 2014a), regulates miR-143 in gastric cancer (Du et al., 2015), and modulates miR-21-PDCD4 in prostate cancer (Yang et al., 2015).

Quercetin influences various lncRNAs to modulate cell death and survival in cancer. It promotes apoptosis in MCF-7 cells by increasing INXS and decreasing UCA1 lncRNA (Rezaie et al., 2021) and enhances apoptosis in NSCLC through the SNHG7/miR-34a-5p pathway (Chai et al., 2021). It impacts the NEAT1/HMGB1 pathway to reduce apoptosis and damage in CI-AKI therapy (Luo et al., 2022) and inhibits epithelial-to-mesenchymal transition (EMT) in prostate cancer by downregulating MALAT1 (Lu *et al.*, 2020), while paradoxically, it induces apoptosis by increasing MALAT1 in rheumatoid arthritis (Pan et al., 2016). Quercetin also has antiangiogenic effects in HUVEC cells via MALAT1 and MIAT (Esteghlal et al., 2021), and its metabolite, isorhamnetin, targets LncRNA-RP11-773H22.4 in T2DM (Matboli et al., 2021).

Diet, therefore, plays a fundamental role in health and disease prevention, especially through phytochemicals found in foods. Research indicates that curry spices, red grapes, soy, and blueberries may aid in cancer prevention (Jasiński et al., 2013; Li et al., 2014b). These dietary components can shape the epigenetic landscape by altering miRNA and lncRNA expression. Moreover, food-derived exosomes can exert therapeutic effects on distant organs. Compounds like sulforaphane in broccoli, for instance, may suppress prostate cancer by modulating lncRNA expression (Beaver et al., 2017; Ekine-Afolabi *et al.*, 2020).

Understanding the interplay between nutrition, dietary factors, and cancer risk through epigenetic modifications unveils significant potential for dietary interventions in cancer prevention and treatment. Research has demonstrated that specific nutrients and bioactive compounds, such as vitamins, polyphenols, and carotenoids, can influence gene expression and modify epigenetic marks, thereby impacting cancer development and progression. This highlights the critical need for further research to explore these relationships and their clinical implications. The development of personalized prevention and treatment strategies based on an individual’s epigenetic profile and dietary habits holds promise for enhancing the effectiveness of cancer therapies, reducing the risk of disease recurrence, and ultimately improving patient outcomes. By tailoring dietary recommendations and interventions, it is possible to create more targeted and effective approaches to cancer prevention and treatment, leveraging the power of nutriepigenetics to combat this complex and pervasive disease.

## Strength of evidence across nutrients

Evidence supporting the epigenetic roles of nutrients varies considerably across compounds. Some nutrients display robust human-based data. Folate, vitamin B12, and vitamin D show consistent effects on DNA methylation and related pathways in both mechanistic and human studies (Padi et al., 2013; Adaikalakoteswari et al., 2015; Gonzalez-Duarte et al., 2015; Alavian-Ghavanini and Rüegg, 2018; Mahajan et al., 2019; Durán et al., 2021; Escobedo-Monge et al., 2024;). Among other bioactives, resveratrol, EPA, and CoQ10 also present combined preclinical, observational, and interventional evidence, particularly regarding miRNA modulation, inflammation, energy metabolism, and mitochondrial function (Tomé-Carneiro et al., 2013; Alehagen et al., 2017; Meng et al., 2020).

However, even for these better-studied nutrients, methodological heterogeneity limits definitive conclusions. Differences in sample type (blood vs. tissue), epigenetic assays, supplementation doses, and intervention duration frequently hinder comparability across studies. Moreover, most human trials examine short-term changes in epigenetic markers rather than long-term clinical outcomes, limiting causal inference in the context of NCD prevention.

Several compounds exhibit emerging but still limited human relevance, with most findings derived from experimental models and only preliminary indications in humans. This group includes curcumin, EGCG, butyrate, and sodium (Choi et al., 2009; Balasubramanian et al., 2010; Yang et al., 2010; Kotchen et al., 2013; Kronski et al., 2014; Momtazi et al., 2016; Bishop et al., 2017; Li et al., 2017; Long et al., 2017; Hassan et al., 2019; Li *et al.*, 2019; Liu *et al.*, 2019; Ferrero et al., 2021; Liao et al., 2023). Many of these studies rely on supraphysiological concentrations or isolated cellular systems that do not fully reflect human metabolic complexity. Observational studies, when available, often report associations without establishing causality, and results across populations remain inconsistent.

Many other compounds, such as quercetin, equol, apigenin, isorhamnetin, caffeic acid, dioscin, and xylobiose, are currently supported exclusively by preclinical studies reporting changes in DNA methylation, histone modifications, or non-coding RNA regulation (Singh et al., 2015; Lim et al., 2016; Mayo et al., 2019; Reyes-Farias and Carrasco-Pozo, 2019; Salem et al., 2019; Hou et al., 2021; Matboli et al., 2021; Sun et al., 2021; Ding et al., 2023; Zhu et al., 2023). While these findings are valuable for mechanistic exploration, their translational relevance remains uncertain. Key challenges include variability between cell lines and animal models, lack of dose-response validation, and the absence of long-term, well-controlled clinical studies that evaluate both epigenetic modifications and clinically meaningful endpoints in NCDs.

Collectively, the current evidence demonstrates promising but uneven progress across nutrients. The field still faces important challenges, including the need for standardized epigenetic assays, harmonized dietary intervention protocols, larger and more diverse cohorts, and integrated multi-omics approaches capable of linking epigenetic changes to metabolic, inflammatory, and clinical outcomes. Addressing these limitations will be essential to strengthen causal inference, resolve existing controversies, and advance nutriepigenetics toward effective applications in NCD prevention and personalized nutrition.

## Conclusion and future perspectives

The field of nutriepigenetics and nutriepigenomics offers promising opportunities for personalized interventions in the prevention and treatment of complex diseases. Epigenetic mechanisms influenced by nutrition have the potential to shape more effective strategies, particularly in addressing the impact of malnutrition. Malnutrition during critical developmental periods can cause long-lasting epigenetic changes, influencing disease susceptibility later in life. Nutrient deficiencies, such as insufficient intake of vitamins and minerals, can disrupt DNA methylation, histone modifications, and ncRNA expression, affecting gene regulation related to inflammation, immune function, and metabolism. These changes emphasize the need for balanced nutrition to maintain epigenetic stability and prevent chronic diseases. Additionally, malnutrition can worsen existing conditions, creating a feedback loop that complicates disease management.

Although the field has advanced considerably, important methodological limitations persist, including the predominance of preclinical studies, heterogeneous biomarkers, and the lack of longitudinal human cohorts capable of establishing causal relationships between dietary components, specific epigenetic marks, and clinical outcomes. Future research should prioritize standardized epigenetic endpoints, integrate multi-omics data and expand population-based studies using fourth-generation sequencing technologies to capture interindividual and interpopulation variability. Strengthening collaboration among researchers, clinicians, and public health professionals will be essential for translating discoveries in nutriepigenetics and nutriepigenomics into targeted dietary interventions and precision-nutrition strategies capable of reducing the global burden of NCDs.

## Data Availability

No new data were generated or analyzed in the preparation of this review article. All information is based on previously published literature cited in the manuscript.
